# Virus Sensor RIG-I Represses RNA Interference by Interacting with TRBP through LGP2 in Mammalian Cells

**DOI:** 10.3390/genes9100511

**Published:** 2018-10-19

**Authors:** Tomoko Takahashi, Yuko Nakano, Koji Onomoto, Mitsutoshi Yoneyama, Kumiko Ui-Tei

**Affiliations:** 1 Department of Biological Sciences, Graduate School of Science, The University of Tokyo, Tokyo 113-0033, Japan; ttakahas@bs.s.u-tokyo.ac.jp (T.T.); yuko.f.nakano@gmail.com (Y.N.); 2 Division of Molecular Immunology, Medical Mycology Research Center, Chiba University, Chiba 260-8673, Japan; konomoto@chiba-u.jp (K.O.); myoneyam@faculty.chiba-u.jp (M.Y.); 3 Department of Computational Biology and Medical Sciences, Graduate School of Frontier Sciences, The University of Tokyo, Chiba 277-8561, Japan

**Keywords:** RNA interference, RLRs, RIG-I, LGP2, TRBP, virus sensor, dsRNA

## Abstract

Exogenous double-stranded RNAs (dsRNAs) similar to viral RNAs induce antiviral RNA silencing or RNA interference (RNAi) in plants or invertebrates, whereas interferon (IFN) response is induced through activation of virus sensor proteins including Toll like receptor 3 (TLR3) or retinoic acid-inducible gene I (RIG-I) like receptors (RLRs) in mammalian cells. Both RNA silencing and IFN response are triggered by dsRNAs. However, the relationship between these two pathways has remained unclear. Laboratory of genetics and physiology 2 (LGP2) is one of the RLRs, but its function has remained unclear. Recently, we reported that LGP2 regulates endogenous microRNA-mediated RNA silencing by interacting with an RNA silencing enhancer, TAR-RNA binding protein (TRBP). Here, we investigated the contribution of other RLRs, RIG-I and melanoma-differentiation-associated gene 5 (MDA5), in the regulation of RNA silencing. We found that RIG-I, but not MDA5, also represses short hairpin RNA (shRNA)-induced RNAi by type-I IFN. Our finding suggests that RIG-I, but not MDA5, interacts with TRBP indirectly through LGP2 to function as an RNAi modulator in mammalian cells.

## 1. Introduction

During virus infection, double-stranded RNAs (dsRNAs), which are generated as viral replication intermediates, are recognized by virus sensor proteins [1,2]. One of the Toll like receptor family proteins (TLRs), TLR3 [3], and retinoic acid-inducible gene I (RIG-I)-like receptors (RLRs) function as virus RNA sensor proteins in endosome and cytoplasm of mammalian cells, respectively [4,5]. RLRs include three proteins, RIG-I, melanoma-differentiation-associated gene 5 (MDA5), and laboratory of genetics and physiology 2 (LGP2). RIG-I and MDA5 are activated by different types of viral RNAs; RIG-I is activated by 5′-triphosphate- or 5′-diphosphate-containing RNA or small RNA duplexes [6,7,8,9,10]; and MDA5 is activated by long dsRNA duplexes [11]. Activated virus sensors transfer the signal to downstream molecules, leading to the production of type-I interferon (IFN). Secreted IFN induces hundreds of IFN-stimulated genes including TLR3 and RLRs to establish potent antiviral states of the cells. Both RIG-I and MDA5 have caspase recruitment domains (CARDs), which are necessary for signal transfer to downstream molecules [1,2]. However, LGP2 does not have CARD, thus the role of LGP2 is controversial. Previous reports show positive or negative effects of LGP2 on RLR signaling. LGP2 positively regulates RIG-I- and MDA5-mediated antiviral responses [12], whereas LGP2 negatively regulates RIG-I-mediated dsRNA recognition [13,14,15]. Furthermore, type I IFN production is promoted in Lgp2^−/−^ mice in response to polyI:C stimulation and vesicular stomatitis virus (VSV) infection, whereas its response to encephalomyocarditis virus (EMCV) is suppressed [16].

RNA silencing or RNA interference (RNAi) is a posttranscriptional gene silencing mechanism conserved in wide variety of organisms, directed by microRNAs (miRNAs), a class of approximately 22-nucleotide (nt)-long non-coding RNAs. Human genome encodes approximately 2000 miRNAs, which regulate gene expression patterns of multiple genes [17]. Many reports demonstrate that mutation or deletion of one miRNA induces serious diseases, indicating the importance of gene regulation by miRNAs [18,19,20]. Primary miRNA transcripts (pri-miRNAs) are cleaved into miRNA precursors (pre-miRNAs) by an endoribonuclease, Drosha, in the nucleus. The pre-miRNAs are exported into the cytoplasm by exportin-5 [21,22], and further processed by Dicer, another endoribonuclease, to yield dsRNA with 2-nt 3′-overhangs [23]. The trans-activation-responsive region (TAR)-RNA binding protein (TRBP) is an RNA silencing enhancer, and functions to recruit pre-miRNA into Dicer [24,25]. TRBP was originally identified as a protein that interacts with the human immunodeficiency virus (HIV)-1 TAR RNA [26] and was later demonstrated to regulate IFN-induced protein kinase R (PKR), a kinase involved in the cellular response to viral infection [27,28]. Small interfering RNA (siRNA) is another type of small non-coding RNA derived from long dsRNA that functions in a similar manner to miRNA. Long dsRNA induces RNAi or RNA silencing in plants and invertebrates [29,30], but activates IFN response in mammals [1,2]. Small RNA duplexes are loaded onto the Argonaute (AGO) protein in the RNA-induced silencing complex (RISC), and unwound into single-stranded RNAs. A guide strand of miRNA/siRNA resides in the RISC represses target mRNAs with sequence complementarities [23,31,32]. 

Recently, LGP2 has been shown to inhibit Dicer-dependent processing of long dsRNA and block mammalian RNAi [33], and we and others reported that TRBP interacted with LGP2 but not with RIG-I or MDA5 [34,35]. Furthermore, we revealed that LGP2 interacts with TRBP, by competing for its dsRNA-binding sites [34]. This competitive binding represses the recruitment of TRBP-bound pre-miRNAs into Dicer, resulting in the decrease of mature miRNA levels and subsequent RNA silencing activities. Thus, the endogenous gene expression might be regulated by miRNAs that are well regulated by LGP2–TRBP interaction during viral infection. Komuro et al. further revealed that TRBP increases *Cardiovirus*-triggered interferon promoter activity only when LGP2 and MDA5 are co-expressed but not MDA5 alone [35]. Thus, TRBP is important for *Cardiovirus*-triggered IFN responses mediated by the LGP2–MDA5 interaction, but is not involved in Sendai virus-triggered IFN response mediated by RIG-I. Based on these results, we examined the contribution of RIG-I and MDA5 for the regulation of RNA silencing. Our results revealed that RIG-I, but not MDA5, regulates short-hairpin RNA (shRNA)-induced RNAi when the expression levels were increased by type-I IFN treatment. The immunoprecipitation assays revealed that both RIG-I and MDA5 did not interact with TRBP directly. However, RIG-I, but not MDA5, interacted with TRBP indirectly through LGP2. Thus, our finding suggests that RIG-I may function as an RNAi modulator in collaboration with LGP2 in mammalian cells. 

## 2. Materials and Methods

### 2.1. Cell Culture

Human HeLa wild-type, HeLa RIG-I^−/−^, HeLa LGP2^−/−^, HeLa TRBP^−/−^, and HEK293T cells were cultured in Dulbecco’s Modified Eagle’s Medium (Wako, Osaka, Japan) containing 10% fetal bovine serum (Gibco, Waltham, MA, USA) at 37 °C with 5% CO_2_. 

### 2.2. Oligonucleotides

siRNA oligonucleotides used for knockdown of AGO2, RIG-I, MDA5, or LGP2 were chemically synthesized as dsRNAs (Shanghai GenePharma, Shanghai, China). Guide and passenger strand RNA oligonucleotides for electrophoretic mobility shift assay (EMSA) were chemically synthesized (Proligo, St. Louis, MO, USA), and purified by polyacrylamide gel electrophoresis. The guide strand was labeled with [γ-^32^P] ATP (6000 Ci/mmol; Perkin Elmer, Waltham, MA, USA). Annealing was performed by incubation at 95 °C for 5 min, and gradually decreasing the temperature to 25 °C. The annealing was confirmed using 15% polyacrylamide gel electrophoresis in Tris–borate–EDTA (TBE) buffer, which can discriminate 21-base-pair double-stranded siRNA from 21-nt single-stranded RNA. Almost all RNAs were recovered as dsRNAs. The RNA sequences are shown in Table S1. 

### 2.3. Plasmid Construction

The expression plasmids of N-terminal FLAG- or C-terminal Myc-tagged TRBP (FLAG-TRBP, or TRBP-Myc) were constructed as described previously [34,36,37]. The plasmids encoding N-terminal FLAG-tagged RIG-I or MDA5 were kindly provided from Dr. T. Fujita (Institute for Frontier Life and Medical Sciences, Kyoto University) [38], and the C-terminal FLAG-tagged LGP2 (LGP2-FLAG) was obtained from Addgene (plasmid #27234, Cambridge, MA, USA). The C-terminal Myc-tagged RIG-I, or LGP2 (RIG-I-Myc, LGP2-Myc) was constructed from FLAG-tagged RIG-I or LGP2 expression plasmids by PCR with primers containing Myc-tag sequence. The primer sequences are shown in Table S2.

### 2.4. shRNA-Induced RNAi Activity Assay

Short hairpin RNA (shRNA)-induced RNAi activity was measured with a luciferase reporter assay. A HeLa cell suspension (1.0 × 10^5^ cells/mL) was inoculated in 24-well plates with or without human type I IFN (IFN-α1; Cell Signaling, Danvers, MA, USA) 1 day before transfection. Cells were transfected with 0.5 μg of pGL3-Control vector (Promega, Madison, WI, USA) encoding the firefly *luciferase* gene, 0.1 μg of pRL-SV40 vector (Promega) encoding the *Renilla luciferase* gene, or 5 ng of pSilencer-3.1-H1-puro vector encoding shRNA against the firefly *luciferase* with Lipofectamine 2000 reagent (Invitrogen, Carlsbad, CA, USA). In the RNAi activity assay, we used shRNA, since the Dicer/TRBP complex is necessary for shRNA cleavage but not for siRNA. Specific siRNAs against each gene for the knockdown (25 pmol) were transfected into the cells 1 day before transfection of the pGL3-Control, pRL-SV40, and pSilencer-3.1-H1-puro vectors. Each siRNA sequence against firefly *luciferase*, Ago2, RIG-I, MDA5, or LGP2 was satisfying the conditions of highly functional siRNA sequences demonstrated previously, and designed by siDirect 2.0 [39,40]. siRNA against green fluorescent protein (GFP) was used as control. The transfected cells were lysed with 1 × passive lysis buffer (Promega) 24 h following transfection. Luciferase activity was measured using the Dual-Luciferase Reporter Assay System (Promega, Madison, WI, USA), and the fold change of firefly luciferase activity normalized to *Renilla* luciferase (firefly luciferase activity/*Renilla* luciferase activity) was determined.

### 2.5. Western Blot

The samples were separated by SDS-PAGE and transferred to a polyvinylidene fluoride membrane using the Trans-Blot Turbo Transfer System (Bio-Rad, Hercules, CA, USA). The membrane was blocked for 1 h in Tris-buffered saline-Triton X-100 or Tween 20 (TBS-T; 20 mM Tris-HCl [pH 7.5], 150 mM NaCl, 0.2% Triton X-100, or 0.1% Tween) supplemented with 5% Difco skim milk (Becton, Dickinson and Company, Franklin Lakes, NJ, USA), and incubated with specific antibodies in Can Get Signal immunoreaction enhancer solution (TOYOBO, Osaka, Japan) at 4 °C overnight. Anti-Myc or anti-FLAG (Cell Signaling, Danvers, MA, USA), anti-AGO2 (Wako, Osaka, Japan), and anti-α-tubulin antibodies (ICN/CAPPEL Biomedicals, Santa Ana, CA, USA) were used. Anti-human RIG-I, MDA5, and LGP2 antibodies were generated by immunizing rabbits with a synthetic peptide [41]. The membrane was washed three times with TBS-T, and reacted with HRP-linked anti-rabbit or anti-mouse antibody (GE Healthcare, Chicago, IL, USA) at room temperature for 1 h. After being washed three times with TBS-T, the membrane was incubated with ECL Prime Western Blotting Detection Reagent (GE Healthcare, Chicago, IL, USA) and visualized with the LAS3000 system (Fujifilm, Osaka, Japan).

### 2.6. Quantitative RT-PCR

Total RNA was extracted by RNeasy mini kit (QIAGEN, Hilden, Germany) and treated with DNase I. A half μg of the total RNA was used for cDNA synthesis by Transcriptor High Fidelity cDNA kit (Roche, Basel, Switzerland). Quantitative RT-PCR was performed by FastStart Universal SYBR Green Master (Roche, Basel, Switzerland) by StepOnePlus realtime PCR system (Applied Biosystems, Foster City, CA, USA). The sequences of the used primers are shown in Table S3.

### 2.7. Electrophoresis Mobility Shift Assay (EMSA)

Purification of the recombinant RIG-I, MDA5, and LGP2 proteins was performed as described previously [8,14]. EMSAs were performed in binding buffer (20 mM Tris [pH 8.0], 1.5 mM MgCl_2_, 50 mM NaCl, 1.5 mM DTT, 100 ng/μL sonicated salmon sperm DNA, 5% glycerol, and 0.4 U/mL RNasin [Promega, Madison, WI, USA]). Each purified protein (3 μM) was incubated with 0.5 nM siRNA containing ^32^P-labeled guide strand for 30 min on ice. The samples were electrophoresed on a 9% polyacrylamide gel in 0.25 × TBE buffer. For quantification, gels were exposed to a Fuji imaging plate and scanned with a Typhoon image analyzer (GE Healthcare, Chicago, IL, USA). 

### 2.8. Immunoprecipitation

A HEK293T cell suspension (2 × 10^6^ cells/dish) was plated into a 6-cm dish 1 day before transfection, and cells were transfected with each combination of the plasmids. Cells were washed with PBS and lysed in cold lysis buffer (50 mM, Tris-NaOH [pH 8.0], 150 mM NaCl, 1 mM Na_3_VO_4_, 1% NP-40, and complete protease inhibitor) 48 h following transfection. For immunoprecipitation, the cell lysate was mixed with 30 µL of Protein G Dynabeads (Life Technologies, Carlsbad, CA, USA) and rotated at 4 °C for 2 h with 2.5 µg of mouse anti-FLAG (Sigma, St. Louis, MO, USA) or mouse anti-Myc antibody (Calbiochem, Burlington, MA, USA), or 2.5 µg of mouse IgG (Santa Cruz Biotechnology, Dallas, TX, USA) as a negative control. The cell lysates were then added to the antibody-bound Protein G Dynabeads and rotated at 4 °C for 2 h. The beads were washed three times with the lysis buffer. To elute the bound proteins, 60 µL of 2 × SDS-PAGE sample buffer was added, and the beads were boiled for 5 min. 

## 3. Results

### 3.1. RIG-I and LGP2 Repress shRNA-Induced RNAi in Human Cells

**Figure 1:**
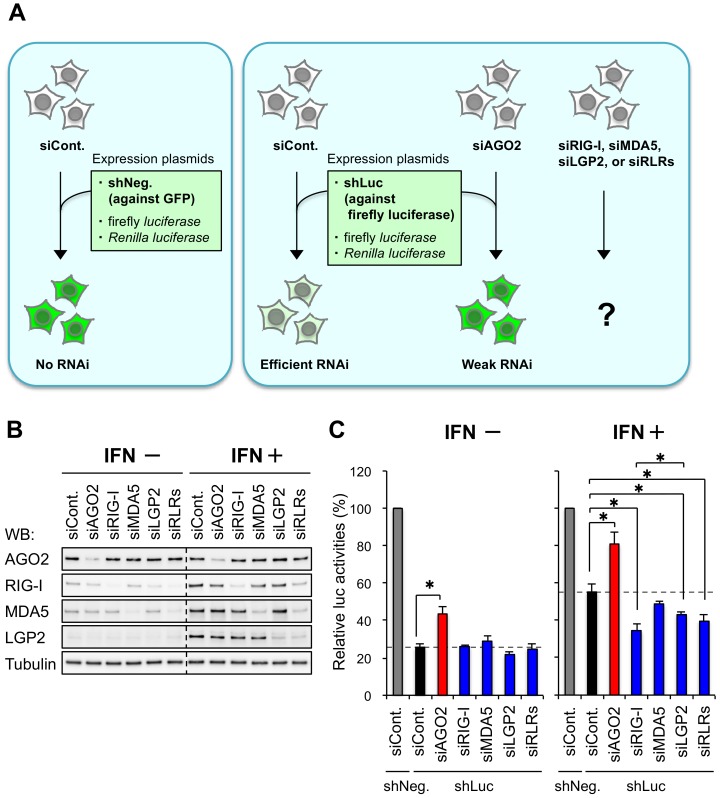
Retinoic acid-inducible gene I (RIG-I) and laboratory of genetics and physiology 2 (LGP2) repress short hairpin RNA (shRNA)-induced RNA interference (RNAi) in human HeLa cells. (**A**) Experimental scheme of shRNA-induced RNAi activity assay. Plasmids encoding shRNA against GFP (shNeg.) or firefly luciferase (shLuc) was transfected with plasmids encoding firefly luciferase and Renilla luciferase into the cells knocked down each gene by small interfering RNA (siRNA) against either of Argonaute 2 (AGO2), RIG-I, melanoma-differentiation-associated gene 5 (MDA5), LGP2, or RIG-I like receptors (RLRs) (mixture of siRNAs against RIG-I, MDA5, LGP2). (**B**) Western blot of AGO2, RIG-I, MDA5, LGP2, and control tubulin in interferon (IFN) non-treated or treated HeLa cells following transfection of specific siRNA against AGO2, RIG-I, MDA5, LGP2, or RLRs. siRNA against GFP was used as control (siCont.). (**C**) Luciferase reporter assay in wild-type HeLa cells with or without IFN. Relative luciferase (luc) activities compared with shNeg. transfection are shown. siRNA against each gene was transfected one day ahead of transfection of shLuc to abolish RNA-induced silencing complex (RISC) saturation. Representative data from two biological repeats, with mean and standard deviation of technical triplicates. *P*-values were determined by the Student’s *t*-test (* *p* < 0.05).

LGP2, one of the RLRs, represses RNA silencing in human cells by preventing the function of TRBP by direct interaction [34]. To investigate the contribution of other RLRs, RIG-I and MDA5, for the regulation of RNA silencing, we performed shRNA-induced RNAi activity assay in human HeLa cells knocked-down each gene by each of specific siRNAs (siAGO2, siRIG-I, siMDA5, and siLGP2) (Figure 1A). AGO2 is an essential protein in the RNAi pathway and used as a positive control [23,31,32]. The siRNA against GFP was used as a control siRNA (siCont.). The mRNA level of AGO2 decreased to 40–50% with siAGO2 transfection, and those of all the other genes decreased to 20–40% (Figure S1), and Western blot showed that the protein levels were substantially repressed with or without IFN treatment (Figure 1B). IFN treatment increased the protein levels of RLRs (RIG-I, MDA5, and LGP2), but not RNAi factor (AGO2) and control (tubulin) (Figure 1B), as reported previously [34]. We then performed a luciferase reporter assay to measure RNAi activity. RNAi activity was measured with a luciferase reporter assay, in which a plasmid expressing shRNA against the firefly luciferase gene was transfected into the cells with the pGL3-control plasmid expressing the firefly luciferase gene, and the pRL-SV40 plasmid expressing the Renilla luciferase gene as an internal control for normalization. RNAi activity was shown as the relative luciferase activity (firefly luciferase activity/Renilla luciferase activity) compared to the control cells transfected with a plasmid expressing shRNA against GFP (shNeg., gray bars in Figure 1C). First, IFN treatment repressed RNAi activities (compare black bar of IFN+ with that of IFN− in Figure 1C), as reported previously [34,42]. Knockdown of AGO2 repressed RNAi activity regardless IFN treatment when compared to control cells (compare black and red bar in each panel of Figure 1C), indicating that AGO2 positively regulates RNAi irrespective of IFN treatment (Figure 1C). Contrary to the knockdown of AGO2, that of RIG-I, LGP2, or RLRs (simultaneous knockdown of RIG-I, MDA5, and LGP2) in IFN-treated HeLa cells increased RNAi activity. However, the knockdown of MDA5 did not increase RNAi activity. No significant effect was observed in the cells knocked down RIG-I, MDA5, LGP2, or RLRs in the absence of IFN treatment (Figure 1C). Thus, upregulation of RIG-I expression by IFN treatment also repress RNAi activity, in addition to LGP2 (Figure 1C).

### 3.2. The Repression of shRNA-Induced RNAi by RIG-I Is TRBP-Dependent

We recently reported that LGP2 represses RNA silencing activity by interacting with the RNA silencing enhancer, TRBP, which recruits pre-miRNA into Dicer [34]. Our previous immunoprecipitation assay revealed that only LGP2, but not RIG-I or MDA5, interacts with TRBP [34]. However, shRNA-induced RNAi activity assay (Figure 1C) showed that RIG-I also represses shRNA-induced RNAi. 

**Figure 2:**
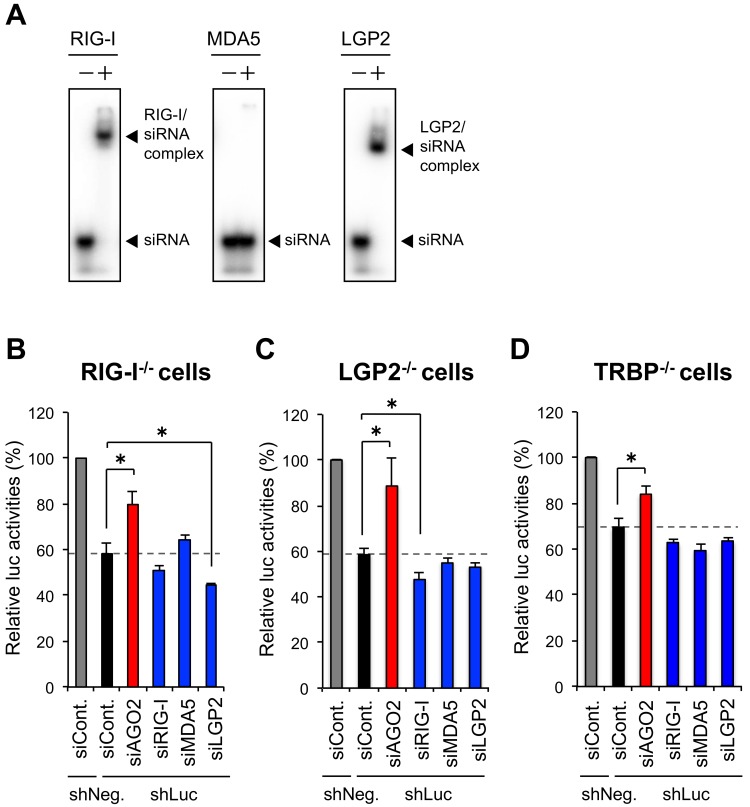
The repression of shRNA-induced RNAi by RIG-I is TAR-RNA binding protein (TRBP)-dependent. (**A**) electrophoresis mobility shift assay (EMSA) of recombinant RLR proteins and siRNA against firefly luciferase (siLuc-36). Each of the purified recombinant RLR proteins and ^32^P-labeled siLuc-36 were incubated and electrophoresed on a 9% polyacrylamide gel. (**B**) Luciferase reporter assay in RIG-I^−/−^ HeLa cells with IFN. (**C**) Luciferase reporter assay in LGP2^−/−^ HeLa cells with IFN. (**D**) Luciferase reporter assay in TRBP^−/−^ HeLa cells with IFN. Representative data from two biological repeats, with mean and standard deviation of technical triplicates. *P*-values were determined by the Student’s *t*-test (* *p* < 0.05).

RIG-I and LGP2 have been shown to bind short virus dsRNAs, while MDA5 does not [8]. Thus, an increase in the amount of RIG-I may sequestrate cellular shRNA and disturb RNAi. An EMSA was performed to examine the interaction of recombinant RLR proteins with 5′-radiolabeled siRNA. Both RIG-I and LGP2 bound the siRNA, while MDA5 did not (Figure 2A). Thus, one possible reason of the repression of RNAi by RIG-I is thought to be a result of siRNA sequestration by these proteins.

To examine whether RNA sequestration is major effect of the repression of RNAi by RIG-I, shRNA-induced RNAi activity assay was performed by the same procedure with Figure 1 in RIG-I^−/−^, LGP2^−/−^, and TRBP^−/−^ cells [34] (Figure S2) established by the clustered regularly interspaced short palindromic repeats (CRISPR)/CRISPR-associated (Cas) genome engineering method [43]. In RIG-I^−/−^ cells, knockdown of LGP2 enhanced RNAi activity significantly, suggesting that RIG-I is not necessary for inhibition of RNAi by LGP2, probably because LGP2 directly interact with TRBP (Figure 2B). However, RIG-I knockdown also enhanced RNAi activity in LGP2^−/−^ cells at marginal but significant level (Figure 2C). TRBP interacts with LGP2 but not RIG-I or MDA5 [34,35], suggesting that RNA sequestration by RIG-I is a possible mechanism by which RIG-I inhibits RNAi. Since TRBP is an RNA silencing enhaner, TRBP^−/−^ cells are assumed to exhibit weaker RNAi activity than wild-type cells, but able to knock down target gene by each siRNA (Figure S3). The knockdown of AGO2 in TRBP^−/−^ cells decreased RNAi activity compared with that of control cells, but knockdown of RIG-I or LGP2 showed no or little effect (Figure 2D). Our previous report revealed that TRBP can bind a limited fraction of miRNAs, whose stem region has tight base-pairing except for the center region, and TRBP can bind to the shRNA used in this experiment [34]. No significant effects were observed by knockdown of RIG-I or LGP2 in TRBP^−/−^ cells, probably because RIG-I and LGP2 also bind to same siRNA (Figure 2A). Thus, the RNA sequestration by RIG-I or LGP2 may not affect RNAi activity in TRBP-KO cells, because RIG-I, LGP2, and TRBP bind to the same shRNA used in this study. These results suggest that RNA sequestration by RIG-I and LGP2 may exhibit marginal effects on RNAi activity in wild type cells, although LGP2–TRBP interaction might be the major inhibitory mechanism of RNAi. 

### 3.3. RIG-I Interacts with TRBP Indirectly through LGP2

**Figure 3:**
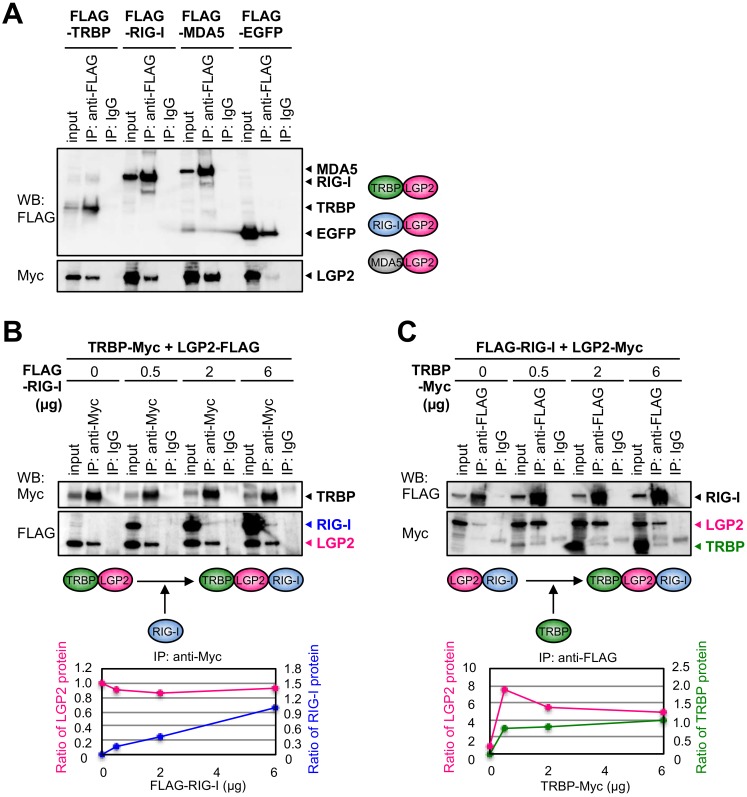

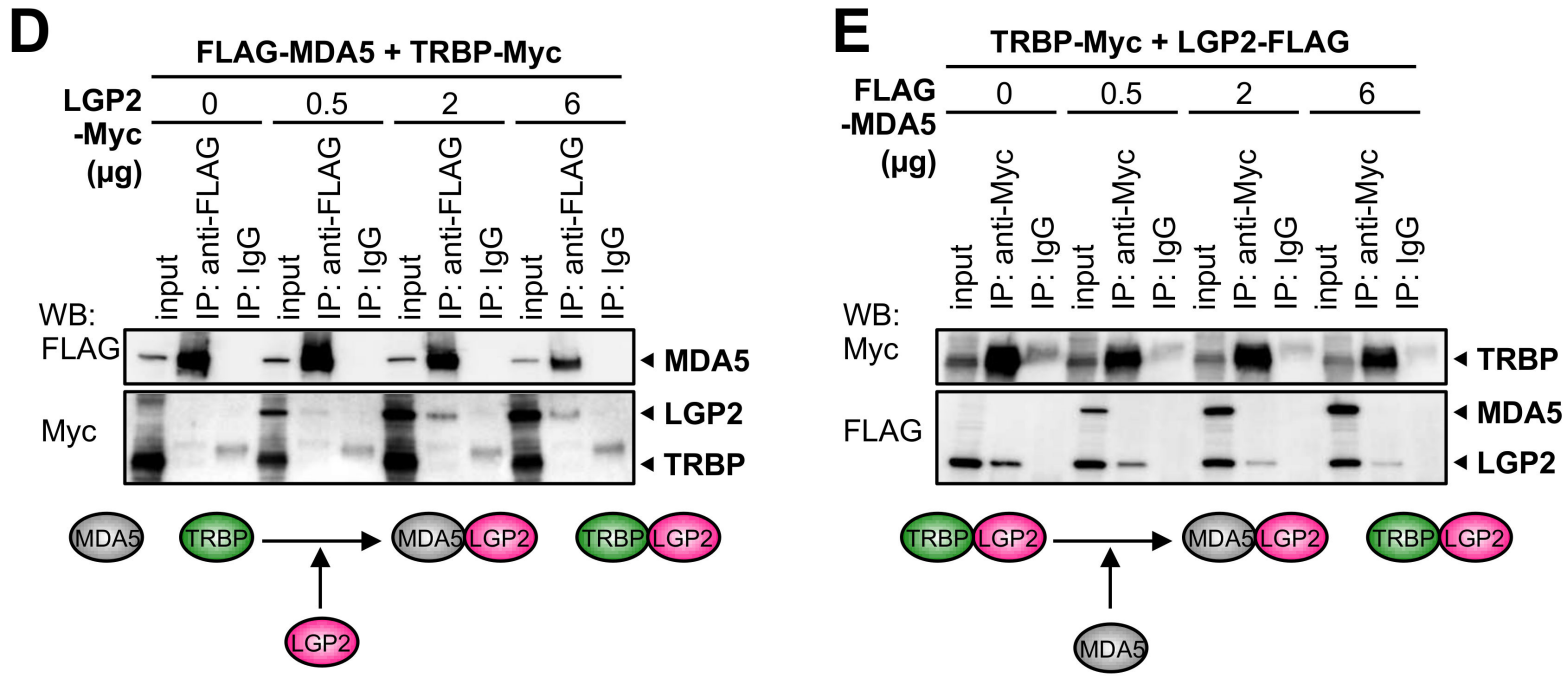
RIG-I interacts with TRBP indirectly through LGP2. (**A**) Immunoprecipitation using HEK293T cells expressing FLAG-TRBP, FLAG-RIG-I, FLAG-MDA5, or FLAG-EGFP with LGP2-Myc protein. TRBP, RIG-I, MDA5, and enhanced green fluorescent protein (EGFP) proteins were immunoprecipitated with anti-FLAG antibody, respectively. LGP2 was detected with anti-Myc antibody. TRBP and EGFP were used as positive and negative controls, respectively. (**B**) Immunoprecipitation using cells expressing TRBP-Myc with LGP2-FLAG, and different concentrations of FLAG-RIG-I. Anti-Myc antibody was used for immunoprecipitation of TRBP, and LGP2 and RIG-I were detected with anti-FLAG antibody. The signal intensities of LGP2 of immunoprecipitate and input, and immunoprecipitated RIG-I were measured using ImageQuant TL (GE Healthcare). The relative ratio of LGP2 signal intensity (immunoprecipitate/input) compared to the control sample without FLAG-RIG-I transfection is shown in pink, and the relative ratio of RIG-I signal intensity compared to the cells transfected with FLAG-RIG-I (6 µg) is shown in blue. (**C**) Immunoprecipitation using cells expressing FLAG-RIG-I with LGP2-Myc, and different concentrations of TRBP-Myc. The relative ratio of LGP2 signal intensity is shown in pink. The relative ratio of TRBP signal intensity is shown in green. (**D**) Immunoprecipitation using cells expressing FLAG-MDA5 with TRBP-Myc, and different concentrations of LGP2-Myc. (**E**) Immunoprecipitation using cells expressing TRBP-Myc with LGP2-FLAG, and different concentrations of FLAG-MDA5. (**B**–**E**) Total amount of empty plasmid and TRBP or each RLR construct was unified at 6 µg in each sample. The presumed results of each protein interaction were shown below each panel.

Our results indicate that RIG-I is also able to inhibit shRNA-induced RNAi (Figure 1C and Figure 2D) without interacting with TRBP directly [34], although RNA sequestration by RIG-I may exhibit the marginal effect on the RNAi activity. However, since LGP2 is able to bind TRBP, it was considered that RIG-I regulates RNA silencing activity by indirect interaction with TRBP through LGP2. Therefore, we investigated whether RIG-I interacts with LGP2 or not in human embryonic kidney 293T cells (HEK293T). FLAG-TRBP, FLAG-RIG-I or FLAG-MDA5 was transfected into the cells with LGP2-Myc and immunoprecipitation was carried out. The results revealed that both RIG-I and MDA5 are able to interact with LGP2, as well as TRBP (Figure 3A). 

Next, to examine the possible indirect interaction of RIG-I with TRBP, we investigated whether TRBP, LGP2, and RIG-I can form triplet complex by immunoprecipitation assay using the expression plasmid of each gene in HEK293T cells (Figure 3B). At first, expression plasmids of TRBP-Myc and LGP2-FLAG were co-transfected with the increased concentrations of FLAG-RIG-I plasmid into the cells. Immunoprecipitation of TRBP protein by anti-Myc antibody revealed that TRBP certainly interacted with LGP2, but also interacted with RIG-I weakly at the increased concentration of FLAG-RIG-I, which can interact with LGP2 but not with TRBP (Figure 3B). Furthermore, FLAG-RIG-I and LGP2-Myc plasmids were co-transfected with the increased concentrations of TRBP-Myc plasmid, and immunoprecipitation of RIG-I protein by anti-FLAG antibody showed that RIG-I interacted with LGP2, and with TRBP weakly at the increased concentration of TRBP-Myc, which can interact with LGP2 but not with RIG-I (Figure 3C). These results suggest that TRBP, LGP2, and RIG-I form protein complex via LGP2, when their expression levels are sufficiently increased. 

Furthermore, to examine the interaction of TRBP and LGP2 complex with MDA5, the expression plasmids of FLAG-MDA5 and TRBP-Myc were co-transfected with the increased concentrations of LGP2-Myc plasmid into HEK293T cells. Immunoprecipitation of MDA5 by anti-FLAG antibody showed that MDA5 certainly interacted with LGP2, but not with TRBP at the any concentration of LGP2-Myc (Figure 3D). The expression plasmids of TRBP-Myc and LGP2-FLAG were co-transfected with the increased concentrations of FLAG-MDA5 plasmid into the cells, and immunoprecipitation of TRBP by anti-Myc antibody showed that TRBP interacted with LGP2, but not with MDA5 at the any concentration of FLAG-MDA5 (Figure 3E). These results suggest that two independent complexes are formed: one consists of MDA5 and LGP2, and the other consists of TRBP and LGP2. Our results of immunoprecipitation in HEK293T cells using overexpressed proteins proposed the possibility that RIG-I may repress shRNA-induced RNAi by inhibiting TRBP function through LGP2, but MDA5 does not.

## 4. Discussion

We recently reported that one of the RLRs, LGP2, interacts with the RNA silencing enhancer, TRBP, to repress its function [34]. TRBP functions to recruit TRBP-bound pre-miRNA into Dicer, resulting enhancement of the miRNA maturation [24,25], and TRBP has greatly higher dsRNA binding affinity compared with Dicer [44], indicating the importance of pre-miRNA recruitment by TRBP *in vivo*. LGP2 interacts with dsRNA-binding sites of TRBP competitively with dsRNA [34]. This competitive binding inhibits the recruitment of pre-miRNA, the miRNA maturation, and subsequent RNA silencing activity of TRBP-bound miRNAs [34]. In this report, we examined the contribution of other RLRs, RIG-I and MDA5, for the regulation of RNA silencing. We showed that IFN treatment attenuated RNAi activity in human cells, and knockdown of RIG-I but not MDA5 enhanced RNAi activity (Figure 1C). IFN treatment increased RLR protein expression (Figure 1B), and upregulated RIG-I repressed RNAi activity by potentiating the effect of LGP2. This may indicate that RIG-I functions to stabilize the interaction between LGP2 and TRBP, although it is unclear why RIG-I depletion enhances RNAi more than LGP2 depletion. Furthermore, we examined the possibility of RNA sequestration by RIG-I for the repression of RNAi. EMSA showed that recombinant RIG-I and LGP2 proteins bound to siRNA, respectively (Figure 2A), as well as TRBP [36]. The shRNA-induced RNAi activity assay in LGP2^−/−^ cells showed that RIG-I knockdown enhanced RNAi activity significantly at marginal level (Figure 2C), suggesting that RNA sequestration by RIG-I is a possible and not negligible mechanism by which RIG-I inhibits RNAi. In TRBP^−/−^ cells, RIG-I did not repress shRNA-induced RNAi (Figure 2D). TRBP can bind a limited fraction of miRNAs, and TRBP can bind to the shRNA used in this experiment [34]. Since RIG-I and LGP2 also bind to same siRNA (Figure 2A), no significant effects by knockdown of RIG-I or LGP2 in TRBP^−/−^ cells might be observed. Thus, RNA sequestration by RIG-I and LGP2 may exhibit modest effects on RNAi activity.

Immunoprecipitation assays in HEK293T cells using expression plasmids revealed that RIG-I interacts with the RNA silencing enhancer, TRBP, indirectly through LGP2. In our previous report, we performed immunoprecipitation assay in HeLa cells to detect endogenous RLR proteins interacting with TRBP, and identified that LGP2 interacts with TRBP directly [34]. In this report, we transfected the expression plasmids encoding RIG-I, MDA5, and LGP2 into HEK293T cells to increase their protein levels sufficiently to detect the indirect interaction with TRBP clearly. Figure 3A shows that both RIG-I and MDA5 can interact with LGP2, independently. Furthermore, LGP2 interacted with both TRBP and RIG-I at once, but did not interact with TRBP and MDA5 simultaneously (Figure 3B–E). Such distinguishable interactions of RIG-I and MDA5 with TRBP-LGP2 may explain the mechanism that knockdown of RIG-I, but not MDA5, increased shRNA-induced RNAi activity (Figure 1C). During viral infection, RIG-I and MDA5 recognize different types of RNA viruses to produce antiviral cytokines. This may indicate that a specific type of virus can repress RNA silencing activity. However, it is unclear how the inhibitory effect on RNAi is related to the anti-viral response. Further studies are needed to examine whether RIG-I represses endogenous miRNA-mediated RNA silencing such as LGP2 and identify their regulated genes.

In summary, one of viral sensor proteins, RIG-I, repressed shRNA-induced RNAi and the interaction with TRBP-LGP2 may have major effect on the repression of RNAi activity. Our finding suggests that RIG-I may function as an RNAi modulator in collaboration with LGP2 and partially through RNA sequestration. These results consolidated our previous report showing the crosstalk between RNA silencing and RLR signaling in mammalian cells. 

## Supplementary Materials

The following are available online at http://www.mdpi.com/2073-4425/9/10/511/s1, Figure S1: Relative mRNA level of AGO2, RIG-I, MDA5, or LGP2 in IFN non-treated or treated cells, Figure S2: Generation of RIG-I knockout HeLa cells (RIG-I^−^/^−^), Figure S3: Confirmation of knockdown at protein level by transfection of siAGO2, siRIG-I, siMDA5, or siLGP2 in TRBP^−^/^−^ cells, Table S1: siRNA sequences used for gene knockdown and EMSA, Table S2: PCR primers used in this study for plasmid construction, Table S3: PCR primers used in this study for qRT-PCR.
