# Supplementary material for: Virus Sensor RIG-I Represses RNA Interference by Interacting with TRBP through LGP2 in Mammalian Cells
Source: Genes (Basel). 2018 Oct 19;9(10):511. doi: 10.3390/genes9100511 (PMC6210652; doi:10.3390/genes9100511)
Supplement: Supplementary file 1 [file genes-09-00511-s001.zip › genes-368755_SupplementaryFiles/genes-368755_SuppplementaryFigure.docx]

Supplementary Materials for

Virus Sensor RIG-I represses RNA interference by interacting with TRBP through LGP2 in mammalian cells

Tomoko Takahashi, Yuko Nakano, Koji Onomoto, Mitsutoshi Yoneyama and Kumiko Ui-Tei

**Supplementary Figure 1. Relative mRNA level of AGO2, RIG-I, MDA5, or LGP2 in IFN-non treated or treated cells.** The mRNA level of each gene in IFN-non treated or treated cells was measured by quantitative RT-PCR. Change in the expression level of each mRNA (red, blue, purple bar) was normalized with GAPDH mRNA (black bar) in the same cells, and the relative mRNA level in IFN-non treated or treated cells was shown by normalization using the value in the control cells.

**Supplementary Figure 2. Generation of RIG-I knockout HeLa cells (RIG-I^-/-^).**

(**A**) The sgRNA was designed against exon 1 of genomic RIG-I gene. Gray box and arrowhead indicate exons and the predicted cleavage site by Cas9 protein, respectively. (**B**) Sequence of RIG-I gene in the cloned RIG-I^-/-^ cells. Two patterns of mutation were detected. Blue line indicates the complementary region of the used guide RNA. (**C**) Western blot was performed using IFN-treated wild-type HeLa cells transfected with siGFP or siRIG-I, and wild-type HeLa cells and RIG-I^-/-^ cells.

**Supplementary Figure 3. Confirmation of knockdown at protein level by transfection of siAGO2, siRIG-I, siMDA5, or siLGP2 in TRBP^-/-^ cells.**

Western blot of IFN-treated TRBP^-/-^ cells after transfection of each siRNA against AGO2, RIG-I, MDA5, or LGP2. The protein level derived from the knocked down mRNA was sufficiently downregulated.
